# Effects of intensive vs. standard blood pressure control on cognitive function: *Post-hoc* analysis of the STEP randomized controlled trial

**DOI:** 10.3389/fneur.2023.1042637

**Published:** 2023-02-01

**Authors:** Jiali Fan, Jingjing Bai, Wei Liu, Jun Cai

**Affiliations:** ^1^Department of Hypertension, National Center for Cardiovascular Diseases, Fuwai Hospital, Peking Union Medical College and Chinese Academy of Medical Sciences, Beijing, China; ^2^Department of Cardiology, Beijing Jishuitan Hospital, Peking University Fourth Hospital, Beijing, China

**Keywords:** cognitive function, intensive BP treatment, Mini-Mental State Examination, cardiovascular events, standard BP treatment

## Abstract

**Background:**

The STEP (Strategy of Blood Pressure Intervention in the older Hypertensive Patients) trial showed that intensive systolic blood pressure (SBP) control resulted in a lower incidence of cardiovascular events than standard treatment. This study analyzed the effects of intensive SBP lowering on cognitive function.

**Methods:**

STEP was a multicenter, randomized controlled trial of hypertensive patients aged 60–80 years. Participants were randomly assigned (1:1) to SBP goals of 110–130 mmHg (intensive treatment) or 130–150 mmHg (standard treatment). Each individual was asked to complete a cognitive function test (Mini-Mental State Examination; MMSE) at baseline and during follow-up. The primary outcome for this study was the annual change in MMSE score. Subjects with a score less than education-specific cutoff point were categorized as cognitive decline.

**Results:**

The analysis enrolled 6,501 participants (3,270 participants in the intensive-treatment and 3,231 participants in the standard-treatment groups). Median follow-up was 3.34 years. There was a minor change in MMSE score, with an annual change of −0.001 [95% confidence interval [CI] −0.020, 0.018] and 0.030 (95% CI 0.011, 0.049) in the intensive- and standard-treatment groups, respectively (*p* = 0.052). Cognitive decline occurred in 46/3,270 patients (1.4%) in the intensive-treatment group and 42/3,231 (1.3%) in the standard-treatment group (hazard ratio 0.005, 95% CI 0.654, 1.543, *p* = 0.983).

**Conclusions:**

Compared with standard treatment, intensive SBP treatment did not result in a significant change in cognitive function test score. The impact of intensive blood pressure lowering was not evident using this global cognitive function test.

**Trial registration:**

ClinicalTrials.gov. Unique identifier: NCT03015311.

## Introduction

The societal and financial burden of dementia and other forms of cognitive impairment is increasing, and there is a growing body of research that aims to determine the modifiable risk factors that can prevent or postpone decline in cognitive function (1). Previously, observational studies have shown that cardiovascular disease and its risk factors are associated with a higher risk for cognitive decline (2). Hypertension is a major public health challenge affecting over one billion people worldwide (3, 4), and it has been identified as a potentially modifiable risk factor by the Lancet Commission on dementia prevention, intervention, and care (5). The National Academies of Sciences, Engineering, and Medicine evaluated current evidence and distinguished blood pressure (BP) management as an effective intervention for preventing cognitive decline (2). A meta-analysis of randomized clinical trials showed that, compared with controls, BP lowering with antihypertensive agents was significantly associated with a lower risk of incident dementia or cognitive decline (7.0 vs. 7.5%, odds ratio 0.93, 95% confidence interval [CI], 0.88–0.98) (6).

Moreover, recent hypertension practice guidelines have advocated lower BP targets for high-risk patients, since intensive BP control provided additional cardiovascular benefits than standard regimens (7, 8). Randomized controlled trials provide inconclusive evidence on intensive BP interventions in hypertensive patients for delaying or slowing of cognitive decline, preventing mild cognitive impairment (MCI), or dementia. Some of the most encouraging evidence comes from the Systolic Blood Pressure Intervention Trial (SPRINT) (9), which aimed to determine whether intensive BP control (< 120 mmHg) would reduce cardiovascular events compared with standard treatment (< 140 mmHg). The SPRINT-MIND study was designed to determine if intensive BP control vs. standard treatment changed the risk of probable dementia and MCI (10). Data revealed that intensive BP control did not result in a significant reduction in the risk of probable dementia during a median follow-up of 5.11 years. Although there was a reduced incidence of MCI (hazard ratio [HR], 0.81, 95% CI, 0.69–0.95) and of a combined MCI and dementia outcome (HR, 0.85, 95% CI, 0.74–0.97).

The aim of this study was to evaluate the impact of intensive SBP management on cognitive function, using a cognitive function test during the follow-up period of the Strategy of Blood Pressure Intervention in the Elderly Hypertensive Patients (STEP) study.

## Materials and methods

### Study participants

The design and methods of STEP (11) have been described previously. Briefly, this multicenter, randomized clinical trial implemented at 42 clinical centers throughout China compared the effect of intensive SBP-lowering treatment (target 110–130 mmHg) vs. standard treatment (target 130–150 mmHg) on cardiovascular events in older Chinese participants with hypertension. Participants were aged 60–80 years of age, with SBP 140–190 mmHg or taking antihypertensive medication at baseline. Individuals with a history of ischemic or hemorrhagic stroke, or with a diagnosis of dementia or taking medications for dementia, were excluded. All participants provided written informed consent. This study was approved by the Ethics Committee of all clinical centers.

### Procedures

**Figure 1:**
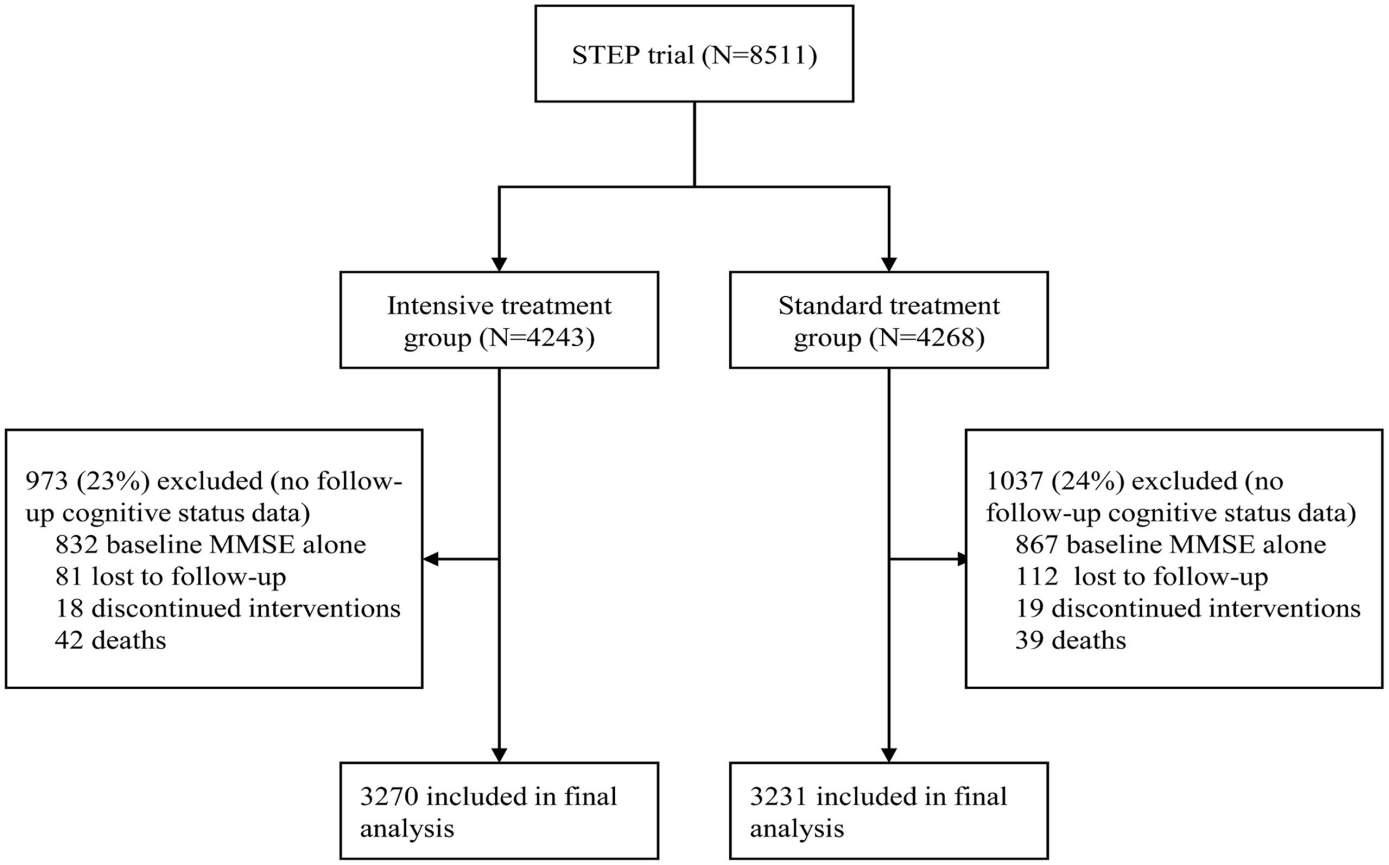
Screening, randomization, and follow-up of participants in this cognitive function analysis.

Enrollment started on January 10 and ended on December 31, 2017. Algorithms for BP intervention and monitoring in STEP have been published previously (detailed information available in the Supplementary Appendix of STEP available at NEJM.org). In general, three major classes of antihypertensive agents, including olmesartan (an angiotensin receptor blocker), amlodipine (a calcium channel blocker), and hydrochlorothiazide were provided free to participants. Following randomization, antihypertensive regimens were adjusted based on office SBP measurements to meet the BP target at baseline and during the entire follow-up period. All participants received a cognitive screening test [Mini-Mental State Examination (12); MMSE] at baseline and years 1, 2, and 3 during follow-up. Cardiovascular events, which was a composite of stroke, acute coronary syndrome, acute heart failure, atrial fibrillation, coronary revascularization, or death from cardiovascular causes, were recorded during follow-up. As of December 31 2020, 343 events had been reported, revealing a clear cardiovascular benefit in the intensive-treatment group. On the advice of the Data and Safety Monitoring Committee, the trial was thus terminated. All participants were assigned the final MMSE test (Figure 1). All the authors take responsibility for the accuracy and integrity of the data. Data in this study will be obtained by other researchers through contacting the corresponding author.

### Cognitive impairment screening instrument

There are many screening instruments available assisting in detecting MCI and dementia. Most instruments have been used in well-designed diagnostic studies or for providing additional quantitative information in randomized clinical trials. The MMSE, which takes 7–10 min to complete, is the most widely used instrument. It comprises seven cognitive domains, including orientation to time and place, attention and calculation, memory, language, and visuospatial abilities. A higher score indicates better cognitive performance. The MMSE has been modified according to sociocultural characteristics and used for epidemiological studies in China (Chinese version of the Mini-Mental Status Examination) (12–14). Previous studies have revealed that level of education plays an essential role in MMSE performance. In particular, illiteracy is still widespread among older people in low- and middle-income countries. Therefore, cut-off points based on the educational background of responders have been widely used: 17/18 for those without formal education, 20/21 for those with primary school education, and 24/25 for participants with middle school or higher education, resulting in a sensitivity and specificity of 85.2 and 92.7%, respectively, for detecting dementia in China (13, 14). In our study, subjects with a score less than education-specific cutoff point were categorized as cognitive decline.

### Outcomes

The primary outcome for this cognitive function analysis was the annual change in MMSE score. The annual change in ΔMMSE score was also reported, with ΔMMSE defined as MMSE score at the first, second and third year and last visit minus the baseline score. We also observed the STEP primary outcome, which was composed by stroke, acute coronary syndrome, acute heart failure, atrial fibrillation, coronary revascularization, or death from cardiovascular causes.

### Statistical analysis

Baseline characteristics were presented as mean (SD) for continuous variables or as counts (percentages) for nominal variables. We compared differences across groups with wilcoxon rank sum tests or chi-square tests. The robust linear mixed model was used to compare longitudinal change in MMSE and ΔMMSE between the treatment groups. The model included the fixed effect of treatment, and random effects of participant and clinic center. The random effect accounted for the effect of longitudinal change of one participant and correlations of participants at the same clinic site. Detailed information about robust linear mixed model was available in the Supplementary material. Analysis of annual change in MMSE and ΔMMSE was based on the assumption of a linear annual slope. For graphics, we also implemented natural spline to the model, which can flexibly model the effect of time. In analyses of the effect of intensive treatment vs. standard treatment on cognitive decline, the Fine–Gray sub-distribution hazard model was used to account for the competing risk of death. The linear mixed-effect model was used to examine the effect of the occurrence of cardiovascular events on the subsequent MMSE test. We conducted subgroup analyses by age, sex, education, marital status, current smoking, current drinking, urbanization, and diabetes mellitus. HRs and 95% CIs were calculated. A two-sided *P* < 0.05 denoted significance. Analyses were performed using R software, version 3.6.3.

## Results

A total of 8,511 patients were included in STEP. Participants unable to undergo follow-up visits included 81 who died, 193 who were lost to follow-up, and 37 who discontinued interventions. One thousand six hundred ninety-nine patients only had baseline MMSE score. After removal of participants without follow-up cognitive data, 3,270 participants were included in the intensive-treatment group and 3,231 in the standard-treatment group in the final analysis. When compared with participants remaining in this cognition analysis, participants without follow-up cognitive assessments were older, had a higher percentage of males, had lower SBP and diastolic BP, had lower cognitive test scores at baseline (Supplementary Table S1). Moreover, participants without follow-up cognitive assessments had a significantly higher incidence of cardiovascular events and all-cause death (Supplementary Table S2). Furthermore, there was no evidence of a differential effect of BP intervention treatment with respect to the incidence of cardiovascular events, all-cause mortality, and renal function between patients in this cognitive function analysis and those without (interaction *p* > 0·05), as shown in Supplementary Table S3.

Baseline characteristics of participants in this analysis are shown in Table 1. Mean age was 66.1 years, with 19% of participants aged ≥70 years. Males comprised 45.6% of participants. Mean SBP at baseline was 146.8 mmHg (standard deviation [SD] 16.8 mmHg) and 146.5 mmHg (SD 16.4 mmHg). Mean MMSE scores were 28.90 (SD 1.92) and 28.92 (SD 1.89) in the intensive- and standard-treatment groups, respectively. There was a sustained mean between-group difference in SBP (Supplementary Figure S1) of 8.6 mmHg (95% CI 8.3, 8.9) from 1 month after randomization to 45 months after randomization, with a mean SBP of 127.5 mmHg (95% CI 127.3, 127.7) in the intensive-treatment group and 136.1 mmHg (95% CI 135.9, 136.3) in the standard-treatment group.

**Table 1:** Baseline characteristics of participants in this cognitive function analysis.

	**Treatment group**		
**Characteristics**	**Standard** **(*****n*** = **3231)**	**Intensive** **(*****n*** = **3270)**	***P*** **value**
Age, mean (SD)	66.18 (4.75)	66.12 (4.83)	0.658
Age≥70 yrs, No. (%)	609 (18.8)	631 (19.3)	0.669
Male, No. (%)	1,461 (45.2)	1,502 (45.9)	0.580
Body-mass index, mean (SD)	25.64 (3.17)	25.58 (3.18)	0.437
SBP, mean (SD), mm Hg	146.50 (16.41)	146.84 (16.80)	0.410
DBP, mean (SD), mm Hg	82.83 (10.58)	83.19 (10.69)	0.171
Smoking, No. (%)	498 (15.4)	531 (16.2)	0.380
Drink, No. (%)	836 (25.9)	839 (25.7)	0.864
Not living alone, No. (%)	3,029 (93.7)	3,087 (94.4)	0.286
Urban, No. (%)	2,996 (92.7)	3,016 (92.2)	0.479
Education^‡^			0.909
1	174 (5.4)	177 (5.4)	
2	589 (18.2)	598 (18.3)	
3	1,080 (33.4)	1,100 (33.6)	
4	1,051 (32.5)	1,034 (31.6)	
5	337 (10.4)	361 (11.0)	
Medical history -no. (%)			
Cardiovascular disease	219 (6.8)	207 (6.3)	0.497
Diabetes mellitus	619 (19.2)	620 (19.0)	0.864
Hyperlipidemia	1,121 (34.7)	1,206 (36.9)	0.070
Estimated glomerular filtration rate, mean (SD), mL/min/1.73 m^2^ ≥ 60, No. (%)	3,181 (98.5)	3,225 (98.6)	0.637
Framingham Risk Score ≥ 15^†^, No. (%)	2,330 (72.1)	2,397 (73.3)	0.416
Use of statins, No. (%)	596 (18.4)	618 (18.9%)	0.662
Use of aspirin, No. (%)	285 (8.8)	286 (8.7)	0.950
No. of antihypertensive agents, mean (SD)	1.39 (0.66)	1.52 (0.69)	< 0.001
MMSE at baseline, mean (SD)	28.92 (1.89)	28.90 (1.92)	0.708
MMSE at baseline, median (IQR)	30 (28, 30)	30 (28, 30)	0.897

SBP, systolic blood pressure; DBP, diastolic blood pressure; IQR, interquartile range; MMSE, Mini-Mental State Examination; SD, standard deviation.

^‡^Education level one to five denotes without formal education, primary school education, middle school education, high school education, college and higher, respectively.

^†^A Framingham risk score of ≥15% represents a higher 10-year risk of cardiovascular disease.

**Figure 2:**
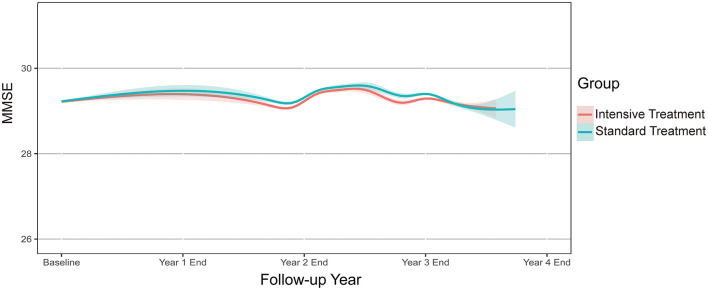
Annual change in MMSE score during follow-up. Solid lines denote estimated mean MMSE for each treatment group based on a robust linear mixed model. Shaded regions indicate 95% confidence intervals. MMSE, Mini-Mental State Examination.

**Figure 3:**
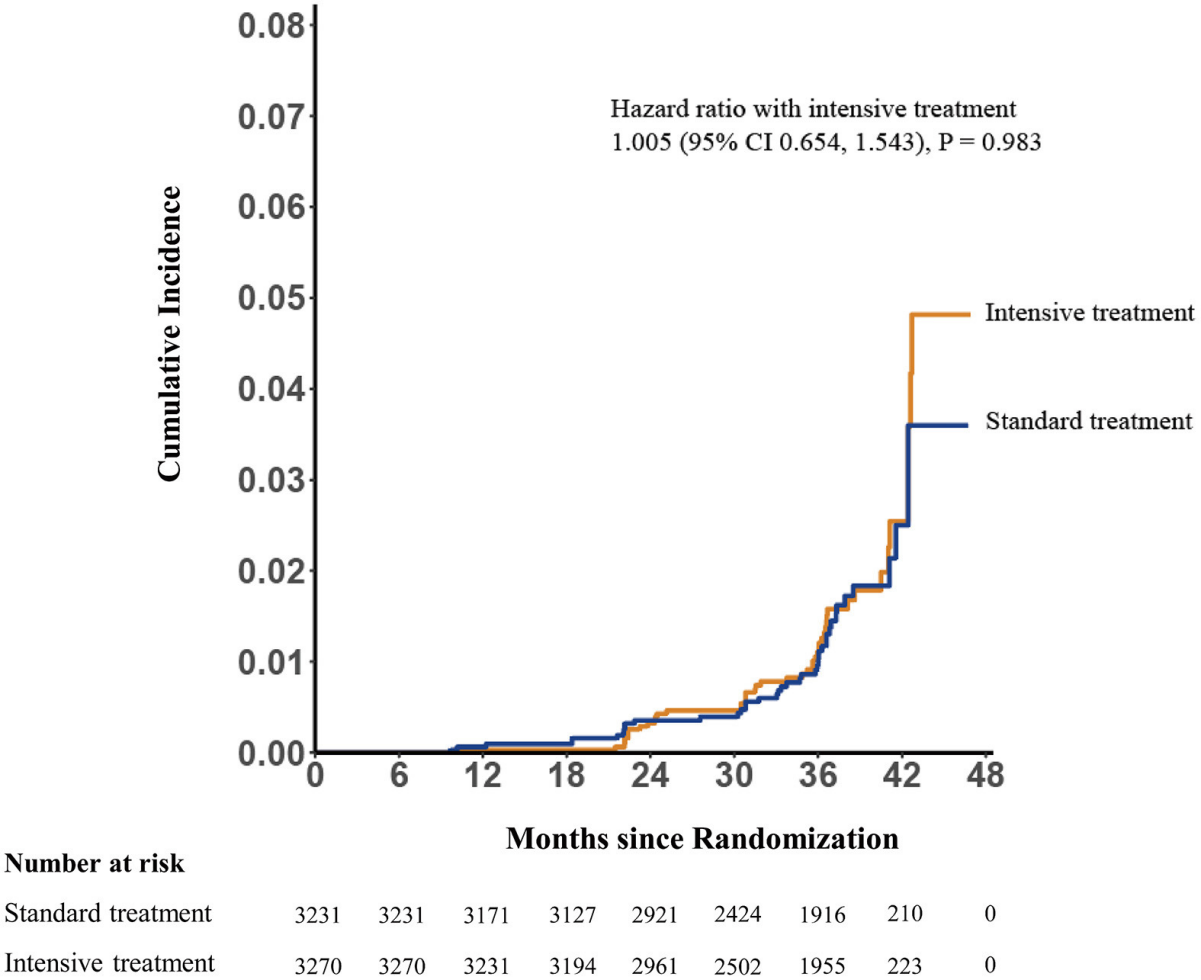
Potential cognitive impairment by treatment group. Hazard ratio 1.005; 95% CI, 0.654, 1.543, *p* = 0.983 for group comparison of incidence.

Mean final MMSE scores were 28.96 (SD 1.87) and 29.08 (SD 1.78) in the intensive- and standard-treatment groups, respectively. Mean ΔMMSE scores were 0.07 and 0.16 in the intensive- and standard-treatment groups, respectively. Figure 2 showed the estimated trajectories for mean MMSE score, estimated using natural spline within a linear mixed model. Over a median follow-up of 3.34 years (interquartile range 3.22–3.51 years) there was very little change in either group, with an annual change of −0.001 (95% CI −0.020, 0.018) and 0.030 (95% CI 0.011, 0.049) in the intensive- and standard-treatment groups, respectively (*p* = 0.052). Annual change in ΔMMSE score between two groups was not significant (*p* = 0.103), with annual slopes of 0.021 (95% CI 0.005, 0.036) and 0.042 (95% CI 0.026, 0.058) for the intensive- and standard-treatment groups, respectively (Table 2). Subgroup analyses revealed that there was no evidence of differential effects (interaction *p* > 0.05) (Table 3). Cognitive decline occurred in 46/3,270 patients (1.4% [0.4% per year]) in the intensive-treatment group, as compared with 42/3,231 patients (1.3% [0.4% per year]) in the standard-treatment group (HR 1.005, 95% CI 0.654, 1.543, *p* = 0.983) (Figure 3).

**Table 2:** Annual change in MMSE score by treatment group^*^.

**Outcome**	**Standard estimate** **(95% CI)**	**Intensive estimate** **(95% CI)**	**Mean difference estimate** **(95% CI)**	***P* value**
Annual change of MMSE	0.030 (0.011, 0.049)	−0.001 (−0.020, 0.018)	−0.031 (−0.058, −0.005)	0.052
Annual change of ΔMMSE	0.042(0.026, 0.058)	0.021 (0.005, 0.036)	−0.021 (−0.043, 0.0002)	0.103

MMSE means Mini-Mental State Examination.

^*^Estimate of annual change of MMSE is performed by using mixed linear regression, assuming linear annual change and adjusting for participant and clinic center.

**Table 3:** Subgroup analysis of annual change in MMSE score^*^.

	**Standard estimate** **(95% CI)**	**Intensive estimate** **(95% CI)**	***P* interaction**
Age			0.263
60–69	0.034 (0.014, 0.054)	0.006 (−0.014, 0.025)	
70–80	0.013 (−0.035, 0.061)	−0.027 (−0.074, 0.020)	
Sex			0.960
Male	0.021 (−0.003, 0.045)	0.004 (−0.020, 0.028)	
Female	0.040 (0.011,0.070)	−0.007 (−0.036, 0.021)	
Education^§^			0.936
< 3	0.114 (0.055, 0.173)	0.141 (0.083, 0.199)	
≥3	0.019 (0.001, 0.037)	−0.023 (−0.041, −0.005)	
Marital status			0.171
Currently living alone	0.060 (−0.023, 0.143)	−0.003 (−0.089, 0.084)	
Not	0.028 (0.009, 0.048)	−0.001 (−0.019, 0.019)	
Urbanization			0.271
Urban	0.023 (0.003, 0.042)	−0.010 (−0.029, 0.010)	
Rural	0.139 (0.083, 0.195)	0.106 (0.055, 0.158)	
Current smoke			0.549
Yes	0.008 (−0.037, 0.053)	−0.013 (−0.055, 0.030)	
No	0.034 (0.013, 0.055)	0.001 (−0.020, 0.022)	
Current drink			0.779
Yes	0.023 (−0.010, 0.055)	0.013 (−0.019, 0.045)	
No	0.033 (0.010, 0.056)	−0.007 (−0.030, 0.016)	
Diabetes Mellitus			0.187
Yes	0.092 (0.046, 0.138)	−0.015 (−0.061, 0.031)	
No	0.017 (−0.004, 0.038)	0.003 (−0.017, 0.024)	

CI, confidence interval; MMSE, Mini-Mental State Examination.

^§^Education < 3 denotes ≤ 6 years education. Education ≥3 denotes >6 years of education.

^*^Estimate of annual change of MMSE is performed by using mixed linear regression, assuming linear annual change and adjusting for participant and clinic center.

During the follow-up period, we identified 83 stroke, 87 acute coronary syndrome, and 215 STEP primary outcome among participants in this cognitive function analysis. In this study, participants with STEP primary outcome had a similar rate of subsequent cognitive decline compared with those without (4/166 vs. 84/6,335, respectively; HR 1.27, 95% CI 0.43, 3.78, *p* = 0.665). However, the occurrence of stroke was associated with a significantly higher rate of subsequent cognitive decline (4/69 vs. 84/6,432, respectively; HR 4.42, 95% CI 1.44, 13.55, *p* = 0.009). The occurrence of STEP primary outcome was associated with a significantly larger decrease in subsequent MMSE score (−0.498, 95% CI −0.745, −0.252, *p* < 0.001). Moreover, there was a significantly larger decrease in subsequent MMSE score (−0.792, 95% CI −1.169, −0.415, *p* < 0.001) for participants with stroke as compared with those without. Supplementary Figure S2 showed the estimated trajectories for mean MMSE score, estimated using natural spline within a linear mixed model between patients with and without stroke events.

## Discussion

In this cognitive function analysis of STEP participants who completed a cognitive function test repeatedly over a median follow-up period of 3.34 years, intensive BP control did not have a significant effect on cognitive function as compared to standard BP control. In general, there was a slight longitudinal change in Mini-Mental State Examination score during follow-up for both treatment groups. These findings did not change substantively in the subgroup analysis. The incidence of cognitive decline was not significantly different between the intensive- and standard-treatment groups.

Our results are largely consistent with prior intensive BP intervention trials, in which cognitive function was compared between BP intervention groups by using several cognitive function tests. In the Prevention of Decline in Cognition after Stroke Trial (PODCAST) (15), intensive (target SBP < 125 mmHg) or guideline (target SBP < 140 mmHg) BP lowering was implemented among patients with recent stroke. The cognitive function tests scores did not differ between two BP treatment groups at the median follow-up period of 24 months. In the Memory in Diabetes (MIND) substudy within Action to Control Cardiovascular Risk in Diabetes (ACCORD) trial (16), cognitive function was compared between intensive (target SBP < 120 mmHg) and standard (target SBP < 140 mmHg) BP lowering intervention in participants with type 2 diabetes mellitus. There was no significant difference in four cognition function tests between two groups. In the Secondary Prevention of Small Subcortical Strokes (SPS3) trial (17), the changes in Cognitive Abilities Screening Instrument (CASI) score over time were not significantly different between two blood pressure control groups (130–149 vs. < 130 mm Hg) among patients with recent symptomatic lacunar infarcts. A substudy of SPRINT trial (18) revealed that there was no evidence that intensive SBP control had a clinically significant effect on memory, language, executive function, or global cognitive function by implementing the screening and extended cognitive batteries at baseline and during follow-up.

A previous study had revealed that lower cerebral perfusion was associated with accelerated decline in cognition and a higher risk of dementia in the older population (19). A recent systematic review had revealed that there was no effect of antihypertensive treatment on cerebral blood flow (20). Treatment of hypertension should not be hindered by concerns about cerebral blood flow.

In this *post-hoc* analysis of STEP trial, loss to follow-up was notable and assessment of intensive blood pressure treatment on cognitive function outcomes may be influenced by dropout. A previous study conducted by Di Bari et al. aimed at evaluating whether cognitive outcomes was biased by differential dropout in the Systolic Hypertension in the Elderly Program (SHEP) trial (21). When subjects who participated in follow-up cognitive assessments were compared to those who did not, assignment to the placebo group predicted missed assessments. Furthermore, when treatment group assignment was combined with age, gender, race, and education in multivariant logistic regression models, it was found to be a significant predictor of a missing cognitive evaluation. We also did the same analysis. However, there was no evidence of a differential effect of treatment group assignment with respect to the occurrence of missed cognitive assessments in univariate or multivariant logistic regression analysis. Moreover, participants without follow-up cognitive assessments had a significantly higher incidence of cardiovascular events and all-cause death. We assumed that the occurrence of cardiovascular events or death influenced patients' status, resulting in the missed cognitive assessment.

Previous studies have reported that antihypertensive medications have varying effects on cognitive function. In STEP trial, all participants were given three primary hypertension medications for free, including Olmesartan (angiotensin receptor blocker [ARB]), Amlodipine (calcium channel blocker [CCB]), and Hydrochlorothiazide. Additional antihypertensive medications, including as β-blockers, were prescribed if physicians thought it necessary and suitable. Patients began treatment with ARB and CCB, and medication regimens were tailored and adjustment to meet the blood pressure target in both groups. We were unable to ascertain whether one type of antihypertensive medicines was superior to the others in terms of cognitive function benefit. It looks probable that the benefits of blood pressure reduction are related to the level of BP achieved rather than the specific medication regimens or antihypertensive medicines class (22). However, a network meta-analysis confirmed the favorable benefits of antihypertensive therapy on cognitive decline and dementia prevention, and also suggest that these effects may vary among medication classes, with ARBs appearing to be the most effective (23). Nonetheless, there is inadequate evidence to identify whether certain antihypertensive classes may be more beneficial than others in terms of cognitive decline and dementia.

The annual rates of change in MMSE in this trial were −0.001 and 0.030 in the intensive- and standard-treatment groups, respectively. In the ACCORD MIND trial, annual change of MMSE were −0.075 and −0.090 in intensive- and standard- BP intervention group, respectively (16). Variations in MMSE responses or trends may result from a difference in version of the test, BP intervention target, and geographic location as well as the cultural and ethnic background of the participants. Moreover, the decline rates were lower than general older Chinese populations. For example, in the Chinese Longitudinal Healthy Longevity Survey (CLHLS), 2,603 participants aged ≥64 years were followed up from 2005 to 2014. In general, the mean MMSE score in participants aged 64–75 years decreased from 27.91 to 27.32, with an annual change in mean MMSE score of −0.066 (12). One possible explanation for this discrepancy is the education level of participants, since a considerable proportion of respondents (50.71%) had no formal education in the CLHLS study. Furthermore, the CLHLS revealed that older people with higher education levels had smaller decreases in cognitive function, which is consistent with studies conducted in other countries (24, 25). Other explanations include socioeconomic factors, lifestyle factors, and cardiovascular risk factors, which have essentially changed and improved over long periods of time in China. Analyses performed using robust linear mixed effect models showed that the mean MMSE score had a slightly increasing trend for participants in the standard BP treatment group, particularly for subjects aged 60–69 years. This improvement might be explained by a learning or practice effect induced by repetition of the test. Another possible interpretation is that BP reduction may cause slightly better cognitive performance in the short term. The OSCAR trial also reported that 6 months of eprosartan therapy was associated with an improvement in the MMSE score in the overall participants (26). The question whether or how such a short-term improvement on a global cognitive test due to drug or lifestyle intervention translates to a long-term cognitive benefit, remains.

In the initial report of STEP trial, intensive BP intervention was associated with a reduced risk of stroke. Evidence from epidemiologic studies has also indicated that stroke and other sub-clinical cerebrovascular disease increase the risk of dementia (2). In this cognitive function analysis, we also found that the occurrence of stroke was associated with a higher rate of cognitive decline and a larger decrease in subsequent MMSE test scores.

Previous observational studies have shown that the effect of hypertension on the subsequent occurrence of cognitive decline or dementia generally depends on age at hypertension diagnosis and the time interval between diagnosis and evaluation of cognitive function (1, 27). Several large, population-based cohort studies have found mid-life hypertension to be associated with accelerated cognitive decline (28, 29) and that prolonged exposure to hypertension increases the risk of dementia (30). The association between late-life hypertension and cognitive function is inconclusive. Some studies reveal that late-life hypertension is associated with worse cognitive performance (31, 32), while others report that high BP is associated with better cognitive function in older patients (33, 34), which may be explained by the inherent limitations of longitudinal studies such as survival bias. Such a result could be misinterpreted as hypertension having a protective effect on cognitive function.

The MMSE is one of the most commonly used cognitive screening tools by both primary care physicians and specialists in western countries (35). The widely used cut-off point of 23/22 or 24/23 has a sensitivity of 89% (95% CI, 0.85 to 0.92) and specificity of 89% (95% CI, 0.85 to 0.93) to detect dementia (36). However, the MMSE might be less reliable for identifying MCI candidates because of inconsistent diagnostic accuracy in different studies, ranging from 0.20 to 0.93 for sensitivity, and 0.48 to 0.93 for specificity (36–38). As a brief screening test, it should not be used as a diagnostic tool to adjudicate mild cognitive impairment. Nevertheless, the MMSE test fulfills its original purpose of providing an objective standardized method to evaluate cognitive function and to document changes over time (39).

The novelty of the study is the examination of intensive blood pressure treatment and cognitive function in a sample of Chinese adults, which has not been widely studied. Although intensive blood pressure control has been demonstrated to improve cardiovascular outcomes in older individuals in several large trials, the ideal systolic blood pressure target is still unknown. STEP trial provides crucial proof that, in older Chinese hypertensive patients, a systolic blood pressure target of 110 to < 130 mm Hg was associated with favorable cardiovascular outcomes. What's more, the association between intensive blood pressure treatment and cognitive performance remains debatable among older hypertensive patients. Our study using a global cognitive function test, MMSE test, which was implemented in STEP trial at baseline and during follow-up period, to monitor the cognitive function.

Our study has following strengths. First, STEP has a large sample size of Chinese older participants. The large sample size ensures a wide range of social culture and education status. Previous studies show that high education level is a protective factor for cognitive function and older people with higher education levels had smaller decreases in cognitive function. Education level and other sociocultural status, including marital status and urbanization, are considered and discussed in the subgroup analyses. What's more, intervention studies aiming to reduce dementia risk through blood pressure control have extra difficulties in the presence of comorbid conditions that themselves may be risk factors for dementia, such as diabetes. Compared with SPRINT (9), our study includes those patients with diabetes and are discussed in the subgroup analyses. In SPRINT, office blood pressure was measured with the use of an automated system, and trial staffs were not present when the measurement was taken. In the STEP trial, office blood pressure was monitored by qualified trial staffs and home blood pressure monitoring was implemented throughout the study period. What's more, the majority of studies examining the effect of intervention on cognitive function in Chinese patients are either cross-sectional or longitudinal, thus requiring adjustment for many established and potential risk factors. The randomized clinical trials are the gold standard for evaluating intervention efficacy and can better solve this problem by balancing unmeasured or unknown confounding factors. Finally, we used a robust linear mixed effect model that can deal with the clustering issues in the repeated measures data, thus yielding the most unbiased statistical results.

This study does have some limitations. First, the length of the intervention and follow-up was relatively short, similar to other contemporary randomized controlled trials. Previous studies suggested that it may take decades for people with dementia to manifest with clinical symptoms. Second, the attrition rate for MMSE test during follow-up period was high. Additionally, validated cognitive impairment ascertainment methods or dementia diagnosis adjudication were lacked in our study. We only implemented a global cognitive function test. MMSE has inherent deficits such as a ceiling effect, likely rendering the test insensitive for detecting subtle cognitive impairments or underlying brain pathology. Although useful in detecting dementia, the MMSE cannot provide detailed information with respect to impairments in specific cognitive domains. Finally, the MMSE may suffer from normal fluctuations in test performance.

In our study, intensive blood pressure treatment can provide additional cardiovascular benefits without deteriorating global cognitive function when measured using MMSE test. Nevertheless, high-strength experimental evidence that address the association between intensive blood pressure intervention with cognitive function is still insufficient, especially trials need to consider several methodological limitations, including enrollment adults across a range of age and ethnic groups, long-term follow-up, incorporation of imaging and laboratory tests, and implement validated cognitive impairment ascertainment methods. European guidelines have highlighted that patients with higher CVD risk should adapt a benefit based tailored treatment strategy in lowering pressure. Further trials are needed to identify optimal BP management strategy for cognitive benefit among different clinical subgroups.

In Chinese older hypertensive patients, intensive BP control does not have a significant beneficial or detrimental impact on global cognitive function when measured using MMSE score.

## Data availability statement

The raw data supporting the conclusions of this article will be made available by the authors, without undue reservation.

## Ethics statement

The studies involving human participants were reviewed and approved by the Ethics Committee of Fuwai Hospital and all other clinical centers (full list is available as a Supplementary file). The patients/participants provided their written informed consent to participate in this study.

## Author contributions

JF: writing the first draft. JB: statistics. WL and JC: conception and supervision. All authors contributed to the article and approved the submitted version.

## Supplementary material

Supplementary Figure S1 Office systolic blood pressure by treatment group. Targets for the intensive- and standard-treatment groups were 110–130 and 130–150 mmHg, respectively. The mean number of medications is the average number of blood-lowing agents administered for each participant during follow-up. I bars denote 95% confidence intervals.

Supplementary Figure S2 Annual change in MMSE score during follow-up. Solid lines denote the mean MMSE score, estimated using natural spline within a linear mixed model between patients with and without stroke events. Shaded regions indicate 95% confidence intervals. MMSE, Mini-Mental State Examination.
