# Supplementary material for: Effects of intensive vs. standard blood pressure control on cognitive function: Post-hoc analysis of the STEP randomized controlled trial
Source: Front Neurol. 2023 Feb 1;14:1042637. doi: 10.3389/fneur.2023.1042637 (PMC9930906; doi:10.3389/fneur.2023.1042637)
Supplement: Supplementary Figure S1 — Office systolic blood pressure by treatment group. Targets for the intensive- and standard-treatment groups were 110–130 and 130–150 mmHg, respectively. The mean number of medications is the average number of blood-lowing agents administered for each participant during follow-up. I bars denote 95% confidence intervals. [file Table_1.DOC]

# Supplementary materials

Modeling of MMSE Score Using Robust Linear Mixed Models

Linear mixed model has been frequently used in longitudinal studies with continuous repeated measures. This model can model data with multiple levels of random variation, also called variance components. It comprises fixed effects and random effects, routinely assuming that the random effects and the within-subject measurement error possess a normal distribution (1). MMSE score is not normally distributed, as it suffers from skewness, floor and ceiling effects due to bounded score ranges. In this scenario, assuming normal distribution may not provide an accurate estimation of between-subject variation. Robust estimation method of linear mixed model is based on the random effects contamination model, which detecting potential contamination on different sources of variability without unnecessary assumptions about the data’s structure (2). Models include the fixed effects of treatment and random effects of participant (ID) and clinic center (Site). Robust estimating equations are derived from the scoring equations by replacing the residuals and spherical random effects with bounded functions, which down-weights terms with a large absolute value and reduces their influence on model estimates (3). We estimate the annual change of MMSE score or ΔMMSE with robust linear mixed models realized by robustlmm package for the R statistical computing environment. The used code in R was described below, where mmse_diff denotes the MMSE score during follow-up minus the MMSE score at baseline, and mmse_diff_year indicates years since randomization. Group denotes treatment group (1 for Intensive Treatment, 2 for Standard Treatment).

rlmer.mmse=rlmer(formula= mmse_diff ~ mmse_diff_year + (1|ID) + (1|Site), rho.sigma.e = psi2propII (smoothPsi, k=2.28), rho.sigma.b = psi2propII (smoothPsi, k=2.28), data=df_lmm %>% filter (Group==1))

Since this model was very complicated, R could not work when we included other covariates in this model.

| Table S1. Characteristics of participants in this cognitive function analysis versus remaining participants in the trial | | | |
| --- | --- | --- | --- |
| Characteristics | Cognitive Function analysis  No.= 6501 | Not in Cognitive Function analysis  No. = 2010 | P value |
| Intensive group, No. (%) | 3270 (50.3) | 973 (48.4) | 0.145 |
| Age, mean (SD) | 66.15 (4.8) | 66.54 (4.9) | 0.002 |
| Age≥70 yrs, No. (%) | 1515 (23.3) | 543 (27.0) | 0.001 |
| Male, No. (%) | 2963 (45.6) | 996 (49.6) | 0.002 |
| Body-mass index, mean (SD) | 25.61 (3.2) | 25.44 (3.2) | 0.030 |
| SBP, mean (SD), mm Hg | 146.67 (16.6) | 144.08 (16.5) | <0.001 |
| DBP, mean (SD), mm Hg | 83.01 (10.6) | 80.76 (10.2) | <0.001 |
| Smoking, No. (%) | 1029 (15.8) | 343 (17.1) | 0.200 |
| Drink, No. (%) | 1675 (25.8) | 566 (28.2) | 0.036 |
| Education* |  |  | 0.011 |
| 1 | 351 (5.4) | 75 (3.7) |  |
| 2 | 1187 (18.3) | 344 (16.6) |  |
| 3 | 2180 (33.5) | 711 (35.4) |  |
| 4 | 2085 (32.1) | 668 (33.2) |  |
| 5 | 698 (10.7) | 222 (11.0) |  |
| Medical history -no. (%) |  |  |  |
| Cardiovascular disease | 426 (6.6) | 114 (5.7) | 0.173 |
| Diabetes mellitus | 1239 (19.1) | 388 (19.3) | 0.832 |
| Hyperlipidemia | 2327 (35.8) | 805 (40.0) | 0.001 |
| Estimated glomerular filtration rate, mean (SD), mL/min/1.73 m^2^ ≥ 60, No. (%) | 6395 (98.4) | 1979 (98.5) | 0. 862 |
| Framingham Risk Score≥15, No. (%)§ | 4986 (76.7) | 1550 (77.1) | 0.720 |
| Use of statins, No. (%) | 1230 (18.9) | 428 (21.3) | 0. 021 |
| Use of aspirin, No. (%) | 692 (10.6) | 200 (10.0) | 0. 397 |
| No. of antihypertensive agents, mean (SD) | 1.45 (0.67) | 1.35 (0.73) | <0.001 |
| Mini-Mental State Examination scores (MMSE) , mean (SD) | 28.91 (1.90) | 28.79 (1.87) | 0.015 |
| Mini-Mental State Examination scores (MMSE) , median (IQR) | 30 (28, 30) | 30 (28, 30) | 0.05 |
| SBP denotes systolic blood pressure. DBP denotes diastolic blood pressure. MMSE means Mini-Mental State Examination. IQR denotes interquartile range.  ^*^Education level one to five denotes without formal education, primary school education, middle school education, high school education, college and higher, respectively.  ^§^A Framingham Risk Score of 15% or higher means a higher 10-year risk of cardiovascular disease. | | | |

| Table S2. Comparing cardiovascular events and death among participants in this cognitive function analysis versus remaining participants in the trial | | | | | |
| --- | --- | --- | --- | --- | --- |
| Outcome | Cognitive Function  analysis  No.= 6501 | | Not in Cognitive Function analysis  No. = 2010 | | P value |
|  | no. of patients  (%) | % with event per year | no. of patients (%) | % with event per year |  |
| Stroke | 83 (1.3) | 0.4 | 36 (1.8) | 0.5 | 0.28 |
| Acute coronary syndrome | 87 (1.3) | 0.4 | 50 (2.5) | 0.7 | 0.04 |
| STEP primary outcome* | 215 (3.3) | 1.0 | 128 (6.4) | 1.9 | <0.001 |
| Death from cardiovascular causes | 14 (0.2) | 0.06 | 29 (1.4) | 0.4 | <0.001 |
| Death from any cause | 52 (0.8) | 0.2 | 81 (4.0) | 1.2 | <0.001 |
| ^*^STEP primary outcome is a composite of stroke, acute coronary syndrome, acute heart failure, atrial fibrillation, coronary revascularization, or death from cardiovascular causes. | | | | | |

| Table S3. Comparing Primary and Secondary Outcomes by treatment group and participation in this cognitive function analysis | | | | | | |
| --- | --- | --- | --- | --- | --- | --- |
| Outcome | Standard Treatment | | Intensive Treatment | | Hazard Ratio | Interaction |
|  | no. of patients (%) | % with event per year | no. of patients (%) | % with event per year | (95% CI) | P value |
| **STEP primary outcome*** |  |  |  |  |  |  |
| Cognitive Function Subgroup | 127 (3.9) | 1.2 | 88 (2.7) | 0.8 | 0.67 (0.51,0.88) | 0.22 |
| Not in Cognitive Function Subgroup | 69 (6.7) | 2.0 | 59 (6.0) | 1.8 | 0.88  (0.61, 1.26) |  |
| **Stroke** |  |  |  |  |  |  |
| Cognitive Function Subgroup | 50 (1.5) | 0.4 | 33 (1.0) | 0.3 | 0.65  (0.41, 1.01) | 0.72 |
| Not in Cognitive Function Subgroup | 21 (2.0) | 0.6 | 15 (1.5) | 0.4 | 0.78  (0.37, 1.64) |  |
| **Acute coronary syndrome** |  |  |  |  |  |  |
| Cognitive Function Subgroup | 54 (1.7) | 0.5 | 33 (1.0) | 0.3 | 0.59  (0.38, 0.92) | 0.39 |
| Not in Cognitive Function Subgroup | 28 (2.7) | 0.8 | 22 (2.3) | 0.7 | 0.81  (0.46, 1.43) |  |
| **Death from cardiovascular causes** |  |  |  |  |  |  |
| Cognitive Function Subgroup | 10 (0.3) | 0.1 | 4 (0.1) | 0.02 | 0.37  (0.11, 1.26) | 0.22 |
| Not in Cognitive Function Subgroup | 15 (1.4) | 0.4 | 14 (1.4) | 0.4 | 0.92  (0.43, 1.98) |  |
| **Death from any cause** |  |  |  |  |  |  |
| Cognitive Function Subgroup | 26 (0.8) | 0.2 | 26 (0.8) | 0.2 | 1.01  (0.59, 1.75) | 0.73 |
| Not in Cognitive Function Subgroup | 39 (3.8) | 1.1 | 42 (4.3) | 1.3 | 1.13  (0.72, 1.79) |  |
|  |  |  |  |  |  |  |
| **Renal outcome — Reduction in eGFR§** |  |  |  |  |  |  |
| Cognitive Function Subgroup | 48 (1.5) | 0.5 | 43 (1.4) | 0.4 | 1.00  (0.99, 1.00) | 0.76 |
| Not in Cognitive Function Subgroup | 13 (1.3) | 0.4 | 12 (1.3) | 0.4 | 1.00  (0.99, 1.01) |  |
| ^*^STEP primary outcome is a composite of stroke, acute coronary syndrome, acute heart failure, atrial fibrillation, coronary revascularization, or death from cardiovascular causes.  ^§^Renal outcome denote ≥30% reduction to <60 ml/min/1.73 m2 in patients without chronic kidney disease at baseline. | | | | | | |


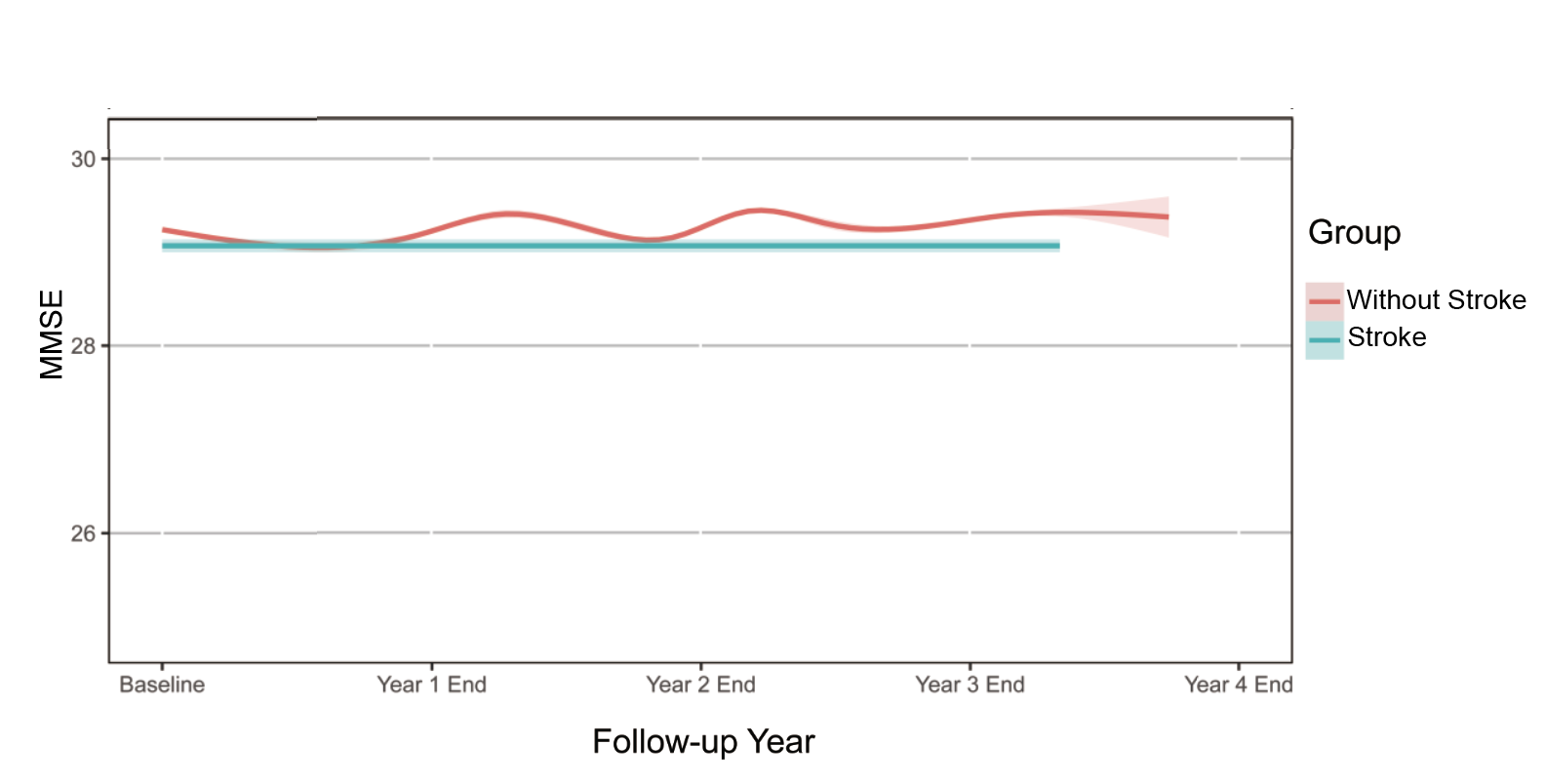


**References**

1. Lachos VH, Dey DK, Cancho VG. Robust Linear Mixed Models with Skew-Normal Independent Distributions from a Bayesian Perspective. *Journal of Statistical Planning and Inference* (2009) 139(12):4098-110.

2. Koller M. Robustlmm: An R Package for Robust Estimation of Linear Mixed-Effects Models. *Journal of statistical software* (2016) 75(1):1-24.

3. Rapp SR, Gaussoin SA, Sachs BC, Chelune G, Supiano MA, Lerner AJ, et al. Effects of Intensive Versus Standard Blood Pressure Control on Domain-Specific Cognitive Function: A Substudy of the Sprint Randomised Controlled Trial. *The Lancet Neurology* (2020) 19(11):899-907.
