# Supplementary material for: Effects of intensive vs. standard blood pressure control on cognitive function: Post-hoc analysis of the STEP randomized controlled trial
Source: Front Neurol. 2023 Feb 1;14:1042637. doi: 10.3389/fneur.2023.1042637 (PMC9930906; doi:10.3389/fneur.2023.1042637)
Supplement: Supplementary Figure S2 — Annual change in MMSE score during follow-up. Solid lines denote the mean MMSE score, estimated using natural spline within a linear mixed model between patients with and without stroke events. Shaded regions indicate 95% confidence intervals. MMSE, Mini-Mental State Examination. [file Table_2.docx]

The STEP trial included Fuwai hospital and other 41 clinical centers (below):

Beijing Hospital;

Peking Union Medical College Hospital;

Beijing Chaoyang Hospital affiliated to Capital Medical University;

The Hospital of Shunyi District;

Beijing Pinggu Hospital;

Lanzhou University Second Hospital;

Guangdong Cardiovascular Institute;

Huizhou Municipal Central Hospital;

The Second Affiliated Hospital to Medical College Shantou University;

Southern University of Science and Technology Hospital;

The First Affiliated Hospital of Guangxi University of Chinese Medicine;

The First Affiliated Hospital of Guangxi Medical University;

Kailuan General Hospital;

The First Affiliated Hospital of Hebei North University;

First Affiliated Hospital of Harbin Medical University;

Hongxinglong Center Hospital;

First Affiliated Hospital of Zhengzhou University;

Zhoukou City Central Hospital;

Renmin Hospital of Wuhan University;

Kang Ya Hospital;

The Second Affiliated Hospital of Baotou Medical College;

Benxi Railway Hospital;

Zhenjiang First People's Hospital;

The Second Affiliated Hospital of Nanchang University;

The 1st Affiliated Hospital of Dalian Medical University;

Qilu Hospital of Shandong University;

Shanghai General Hospital;

Shanxi Bethune Hospital, Shanxi Academy of Medical Sciences;

First Hospital of Shanxi Medical University;

Shanxi Cardiovascular Hospital;

First Affiliated Hospital, Xian Jiaotong University;

West China Hospital (Department of Geriatrics), Sichuan University;

West China Hospital (Department of Cardiovascular Medicine), Sichuan University;

College of Medicine, National Taiwan University;

Pingjin Hospital, Logistics University of PAPF;

The People's Hospital of Ji Xian District;

First Affiliated Hospital of Xinjiang Medical University;

The First People's Hospital of Yinchuan;

Yan'an Hospital Affiliated to Kunming Medical University;

Fuwai Yunnan Cardiovascular Hospital;

The First Hospital of Kunming.
